# Liver and Pancreatic Resection in the Elderly

**DOI:** 10.1155/1997/84160

**Published:** 1997

**Authors:** O. J. Garden

**Affiliations:** University Department of Surgery Royal Infirmary Lauriston Place Edinburgh United Kingdom

## Abstract

*Background*: Liver resection, or pancreaticoduodenectomy, has traditionally been thought to have a high morbidity and. mortality rate among the elderly. Recent improvements in surgical and anesthetic techniques, an increasing number of elderly patients, and an increasing need to justify use of limited health care resources prompted an assessment of recent surgical outcomes.

*Methods*: Five hundred seventy-seven liver resections (July 1985–July 1994) performed for metastatic colorectal cancer and 488 pancreatic resections (October 1983–July 1994) performed for pancreatic malignancies were identified in departmental data bases. Outcomes of patients younger than age 70 years were compared with those of patients age 70 years or older.

*Results*: Liver resection for 128 patients age 70 years or older resulted in a 4% perioperative. mortality rate and a 42% complication rate. Median hospital stay was 13 days, and 8% of the patients required admission to the intensive care unit (ICU). Median survival was 40 months, and the 5-year survival rate was 35%. No difference were found between results for the elderly and those for younger patients who had undergone liver resection, except for a minimally shorter hospital stay fortheyoungerpatients (median, 12 days *vs.* 13 days p=0.003). Pancreatic resection for 138 elderly patients resulted in a mortality rate of 6% and a complication rate of 45%. Median stay was 20 days, and 19% of the patients required ICU admission, results identical to those for the younger cohort. Long-term survival was poorer for the elderly patients, with a 5-year survival rate of 21% compared with 29% for the younger cohort (p=0.03).

*Conclusions*: Major liver or pancreatic resections can be performed for the elderly with acceptable morbidity and mortality rates and possible long-term survival. Chronologic age alone is not a contraindication to liver or pancreatic resection for malignancy.


### Liver and Pancreatic Resection in the Elderly

### ABSTRACT

*Fong, Y., Blumgart, L.H., Fortner, J.G and Brennan, M.F (1995). Pancreatic or liver resection for malignancy is safe and effective for the elderly. Annals of Surgery, 222: 426–437.*

***Background:*** **Liver resection, or pancreaticoduodenectomy, has traditionally been thought to have a high morbidity and mortality rate among the elderly. Recent improvements in surgical and anesthetic techniques, an increasing number of elderly patients, and an increasing need to justify use of limited health care resources prompted an assessment of recent surgical outcomes.**

***Methods:*** **Five hundred seventy-seven liver resections (July 1985–July 1994) performed for metastatic colorectal cancer and 488 pancreatic resections (October 1983–July 1994) performed for pancreatic malignancies were identified in departmental data bases. Outcomes of patients younger than age 70 years were compared with those of patients age 70 years or older.**

***Results:*** **Liver resection for 128 patients age 70 years or older resulted in a 4% perioperative mortality rate and a 42% complication rate. Median hospital stay was 13 days, and 8% of the patients required admission to the intensive care unit (ICU). Median survival was 40 months, and the 5-year survival rate was 35%. No difference were found between results for the elderly and those for younger patients who had undergone liver resection, except for a minimally shorter hospital stay for the younger patients (median, 12 days *vs.* 13 days p=0.003). Pancreatic resection for 138 elderly patients resulted in a mortality rate of 6% and a complication rate of 45%. Median stay was 20 days, and 19% of the patients required ICU admission, results identical to those for the younger cohort. Long-term survival was poorer for the elderly patients, with a 5-year survival rate of 21% compared with 29% for the younger cohort (p=0.03).**

***Conclusions:*** **Major liver or pancreatic resections can be performed for the elderly with acceptable morbidity and mortality rates and possible long-term survival. Chronologic age alone is not a contraindication to liver or pancreatic resection for malignancy.**

*Keywords*: Liver resection, hepatic resection, pancreatic resection, elderly major surgery

### PAPER DISCUSION

In recent years, advances in surgical practice have seen a reduction in morbidity and mortality rates for major hepatobiliary and pancreatic resectional surgery. Given that surgical resection is the only potential curative therapy for malignant disease of the liver and pancreas, many surgeons have advocated a more aggressive approach to the management of such malignancy in the anticipation of demonstrating improved long-term survival. Whilst the title of this paper from the Memorial Sloan-Kettering Cancer Center might support a relaxation of previously stringent selection criteria, the results require close scrutiny before other surgeons rush to follow suit and increase their own resectional practice.

In the management of pancreatic cancer, several recent series testify to the fact that pancreaticoduodenectomy can be undertaken with minimal mortality and low morbidity rates [1,2]. The authors report a creditable 6% mortality rate amongst the 138 patients over the age of 70 years undergoing pancreatic resection. The operative mortality rate was little different than for those patients less than 70 years of age undergoing similar resections. Five year survival was 21%, although none of the 10 patients surviving to five years underwent resection for pancreatic adenocarcinoma. The careful selection of patients for consideration of resection is exemplified by the fact that only 69 of the 138 patients undergoing pancreatic resection had pancreatic adenocarcinoma. The intense demands placed on hospital resource with such surgery is highlighted by the 45% complication rate and the 19% intensive care admission rate, although median hospital stay was 20 days. There are no data available to indicate whether recovery and return to normal activity was different in patients over 70 years of age and, as in similarly reported series, there is no assessment of the quality of life in patients following discharge.

The authors do recognise the potential effect of the pre-referral selection process in contributing to their good results. Further, it is noted that the authors have adopted the use of laparoscopy as a means of avoiding unnecessary laparotomy [3]. Our own experience suggests that a combination of laparoscopy and laparoscopic ultrasonography will avoid unnecessary non-therapeutic laparotomy [4], the morbidity of which is not often appreciated from the publication of selected patients undergoing resectional surgery. The median survival of 18 months following pancreatic resection reported by the authors merely serves to underline the importance of selecting out patients unlikely to benefit from an aggressive surgical approach.

A more encouraging role for hepatic resection in the management of colorectal metastases is evident from a number of studies which have demonstrated five year survival rates of up to 40% [5,6]. The present paper reports an encouraging 35% five year survival rate which is not dissimilar to the 39% five year survival rate observed in patients under the age of 70 years. Forty two percent of patients developed post-operative complications and there was a 4% peri-operative mortality rate. Male patients had a greater risk for complication than female patients and perhaps not surprisingly resection of at least one lobe of the liver and operative times exceeding four hours were associated with increased risk. It is unfortunate that such important factors of post-operative outcome may not therefore be easily predicted before patients are submitted to laparotomy. It is not readily evident from other reported series as to whether patients with other forms of hepatic malignancy can be similarly considered for resectional surgery with advancing years. Operative mortality rates as high as 41% have been reported over the last ten years for patients undergoing resection for primary hepatic malignancy [7,8,9].

It is evident that the authors are indeed able to conclude that "patients with pancreatic or liver malignancy should be considered for surgical therapy regardless of chronologic age". Nonetheless, it is apparent that assessment of individual risk for patients undergoing resectional surgery is not always possible. Relaxation of existing criteria for considering patients for complex hepatobiliary and pancreatic resection should therefore only be undertaken in established centres with a proven track record in this specialist field of surgery.

### *References*

[1] Trede, M., Schwall, G. and Saeger, H. (1990). Survival after pancreaticoduodenectomy. 118 consecutive resections without an operative mortality. *Annals of Surgery*, **211**, 447–458.

[2] Cameron, J.L., Pitt, H.A., Yeo, C.J., Lillemoe, K.D., Kaufman, H.S. and Coleman, J. (1993). One hundred and forty-five consecutive pancreaticoduodenectomies without mortality. *Annals of Surgery*, **217**, 430–435.

[3] Conlon, K.C., Dougherty, E., Kumstra, D.S., Coit, D.G., Turnbull, A.D.M. and Brennan, M.F. (1996). The value of minimal access surgery in the staging of patients with potentially resectable peripancreatic malignancy. *Annals of Surgery*, **223**, 134–140.

[4] John, T.G., Greig, J.D., Carter, D.C. and Garden, O.J. (1995). Carcinoma of the pancreatic head and periampullary region: tumour staging with laparoscopy and laparoscopic ultrasonography. *Annals of Surgery*, **221**, 156–164.

[5] Scheele, J., Stangl, R., Altendorf-Hofmann, A. and Paul, M. (1995). Resection of colorectal liver metastases. *World Journal of Surgery*, **19**, 59–71.
[6] Doci, R., Gennari, L., Bignami, P., Montalto, F., Morabito, A. and Bozzetti, F. (1991). One hundred patients with hepatic metastases from colorectal cancer treated by resection: analysis of prognostic determinants. *British Journal of Surgery*, **78**, 797–801.
[7] Yanaga, K., Kanematsu, T. and Takenaka, K. *et al.* (1988). Hepatic resection for hepatocellular carcinoma in elderly patients. *American Journal of Surgery*, **155**, 238–241.
[8] Fortner, J.G. and Lincer, R.M. (1990). Hepatic resection in the elderly. *Annals of Surgery*, **211**, 141–145.
[9] Nagasue, N., Chang, Y.C. and Takemoto, Y. *et al.* (1993). Liver resection in the aged (seventy years or older) with hepatocellular carcinoma. *Surgery*, **113**, 148–154.

O. J. Garden, MD, FRCS
Senior Lecturer in Surgery
Director of Organ Transplantation
University Department of Surgery
Royal Infirmary, Lauriston Place
Edinburgh
United Kingdom

### Pre-Liver Transplant: Tips Versus Distal Splenorenal Shunt

### ABSTRACT

*Abouljoud, M.S., Levy, M.F., Rees, C.R., Diamond, N.G., Lee, S.P., Mulligan, D.C., Goldstein, R.M., Husberg, B., Gonwa, T.A. and Klintmalm, G.B. (1995)A comparison of treatment with transjugular intrahepatic portosystemic shunt or distal splenorenal shunt in the management of variceal bleeding prior to liver transplantation. Transplantation, 59: 226–229.*

**Recurrent variceal bleeding in liver transplant candidates with end-stage liver disease can complicate or even prohibit a subsequent transplant procedure (OLT). Endoscopic sclero-therapy and medical therapy are considered as first-line management with surgical shunts reserved for refractory situations. Surgical shunts can be associated with a high mortality in this population and may complicate subsequent OLT. The transjugular intrahepatic portosystemic shunt (TIPS) has been recommended in these patients as a bridge to OLT. This is a new modality that has not been compared with previously established therapies such as the distal splenorenal shunt (DSRS). In this study we report our experience with 35 liver transplant recipients who had a previous TIPS (18 patients) or DSRS (17 patients) for variceal bleeding. The TIPS group had a significantly larger proportion of critically ill and Child-Pugh C patients. Mean operating time was more prolonged in the DSRS group ($P$=0.014) but transfusion requirements were similar. Intraoperative portal vein blood flow measurements averaged 2132±725 ml/min in the TIPS group compared with 1120±351ml/min in the DSRS group ($P$<0.001). Arterial flows were similar. Mean ICU and hospital stays were similar. There were 3 hospital mortalities in the DSRS group and none in the TIPS group ($P$=0.1). We conclude that TIPS is a valuable tool in the management of recurrent variceal bleeding prior to liver transplantation. Intraoperative hemodynamic measurements suggest a theoretical advantage with TIPS. In a group of patients with advanced liver disease we report an outcome that is similar to patients treated with DSRS prior to liver transplantation. The role of TIPS in the treatment of nontransplant candidates remains to be clarified.**

